# Influence of the activation mode on long-term bond strength and endogenous enzymatic activity of dual-cure resin cements

**DOI:** 10.1007/s00784-021-04141-x

**Published:** 2021-09-02

**Authors:** Claudia Mazzitelli, Tatjana Maravic, Edoardo Mancuso, Uros Josic, Luigi Generali, Allegra Comba, Annalisa Mazzoni, Lorenzo Breschi

**Affiliations:** 1 Department of Biomedical and Neuromotor Sciences, DIBINEM, University of Bologna, Alma Mater Studiorum, Via San Vitale 59, 40125 Bologna, Italy; 2 Department of Surgery, Medicine, Dentistry and Morphological Sciences, Unit of Dentistry and Oral-Maxillo-Facial Surgery, University of Modena and Reggio Emilia, via del Pozzo 71, 41124 Modena, Italy; 3 Department of Surgical Sciences, Dental School Lingotto, University of Turin, Via Nizza 230, 10126 Turin, Italy

**Keywords:** Resin cement, Dual-cure, Curing mode, Microtensile bond strength, MMPs, In vitro aging, In situ zymography, SEM

## Abstract

**Objective:**

To investigate the long-term microtensile bond strength (µTBS), interfacial nanoleakage expression (NL), and adhesive stability of dual-cure resin cements with/out light activation to dentin.

**Materials and methods:**

Composite overlays (*N* = 20) were luted to deep dentin surfaces with RelyX Ultimate (RXU, 3M) or Variolink EstheticDC (VAR, Ivoclar-Vivadent). A universal adhesive was used for bonding procedures (iBond universal, Heraeus Kulzer). The resin cements were either self-cured (SC; 1 h at 37 °C) or dual-cured (DC; 20s light-cure followed by 15 min self-cure at 37 °C). Specimens were submitted to µTBS immediately (*T*_0_) or after 1 year of laboratory storage (*T*_12_). The fracture pattern was evaluated using scanning electron microscopy (SEM). Data were statistically analyzed with two-way ANOVA/Tukey test. Further, the NL was quantified and analyzed (chi-square test) and in situ zymography was performed to evaluate the endogenous enzymatic activity within the hybrid layer (HL) at *T*_0_ and *T*_12_ (Mann–Whitney test)_._ The significance level for all statistical tests was set at *p* = 0.05.

**Results:**

DC resulted in higher bond strength and decreased fluorescence at the adhesive interface, irrespective of the material and the storage period (*p* < 0.05). Significantly lower bonding performances (*p* < 0.05) and higher endogenous enzymatic activity (*p* < 0.05) were observed within the HL at *T*_12_ compared to *T*_0_ in all tested groups.

**Conclusions:**

Light-curing the dual-cure resin cements, more than the cement materials, accounted for good bonding performances and higher HL stability over time when used with a universal adhesive.

**Clinical significance:**

The curing condition influences the bonding performances of dual-cure resin cements to dentin when used with a universal adhesive.

## Introduction

Resin composite cements have a major role in operative dentistry, yielding enhanced esthetic and mechanical properties of the restoration. They can be classified according to the polymerization mode (self-cure, light-cure, or dual-cure) or based on the dental surface pre-treatment (multi-step or self-adhesive cements). Weakness in the adhesive and cementation procedures, such as incomplete polymerization of the resin cement or reduced adhesive quality, may result in higher water sorption and solubility, increased marginal leakage, and decreased bond strength with consequent clinical failure due to debonding, fractures, or secondary caries [1–5].

There is evidence in the literature that the dual-cure mechanism should be favored for the cementation of indirect restorations as the polymerization reaction continues even in the absence of light, through a synergic combination of self- and light-polymerizing components [1, 4, 5]. However, the interaction between the chemical- and light-activated components makes the dual-curing mechanism very complex. The cross-linked polymer chains formed after immediate light-curing can interfere with the self-cure process due to the entrapment of the chemical polymerization promoters and unreacted monomers into the forming network [6]. After the polymer formation, the viscosity of the material increases. This increase limits the movements of the remaining monomers responsible for additional polymerization [7], reducing the likelihood of bimolecular termination [8]. Moreover, deficiencies in the chemical curing process can increase the density of unreacted double bonds, reduce the polymerization reaction, affect the hardness, and influence the solubility of the cement, which may alter the chemical stability in the oral cavity [9, 10].

The interaction between conventional multi-step dual-cure resin cements and dentin is mediated by the bonding systems, with the universal adhesives representing the latest simplified version. The high concentration of acidic monomers blended into the adhesives [11], and the hydrophilicity of certain adhesives which behave as semipermeable membranes [12] may impair the polymerization reaction and influence detrimentally the bonding performances of adhesively bonded restorations [13]. The interaction between resin cement systems and universal adhesive is still questionable from the aspect of the bonding performances over time [14].

Moreover, although it has been conventionally accepted to preferably select all the materials from the same brand for a single restoration, [15], several manufacturers claim the eclecticism of certain dual-cure resin cements as equally efficient when used in combination with adhesive systems of different brands. This can also have clinical importance, as it is not uncommon for clinicians to use differently branded products, leading to overestimated outcomes.

Accordingly, this in vitro study aimed at evaluating the immediate (*T*_0_) vs 1 year (*T*_12_) microtensile bond strength (µTBS) to dentin, interfacial nanoleakage (NL) expression, and endogenous enzymatic activity within the hybrid layer of dual-cure resin cements with/out light activation. The null hypotheses tested were that light activation (1) does not influence the immediate bonding performances to dentin, (2) does not influence endogenous dentinal enzymatic activity, and (3) does not preserve adhesive interfaces over time.

## Materials and methods

### Microtensile bond strength test

The teeth used in this study were obtained from anonymous individuals following their informed consent under a protocol approved by the Ethical Committee of the University of XXXXXXX (protocol N°: 71/2019/OSS/XXXXXX). Teeth were observed under a stereomicroscope to evaluate the absence of caries, cracks, and fractures. Selected teeth were stored in saline solutions until use, no longer than 1 month.

Twenty sound human third molars were decoronated with a low-speed diamond saw under water cooling (Microremet, Remet, Bologna, Italy) to expose middle/deep dentin. The absence of enamel remnants was assessed under an optical microscope. A standardized smear layer was created on dentin surfaces with #240-grit wet silicon carbide (SiC) paper.

Resin composite build-ups were layered with two 2-mm-thick increments (Venus Diamond, Heraeus Kulzer GmbH, Hanau, Germany, LOT: K010069) compacted into a silicone mold (final height of the restoration: 4 mm). Each increment was polymerized for 40 s using a light-emitting diode curing lamp (LED; Demi™ Plus, Kerr Corp., Orange, CA, USA; light output 1.200-mW/cm^2^, wavelength 450–470 nm) from the top of the restoration. Then, the composite overlays were removed from the mold, and additional 40 s of light irradiations were performed on each side and the bottom of the restoration, previously in contact with the mold. The bonding surfaces of indirect composite resin blocks were wet-polished with #240-grit SiC papers for 30 s. Then, composite disks were cleaned in an ultrasonic bath for 2 min. According to the manufacturer’s instructions, a surface conditioner (iBond Ceramic Primer; Heraeus Kulzer) was brushed on the whole surface of the composite overlay.

The specimens were randomly assigned to 2 groups (*N* = 10) according to the material used for luting procedures: (1) RelyX Ultimate (RXU; 3 M ESPE, Seefeld, Germany); (2) Variolink EstheticDC (VAR; Ivoclar-Vivadent, Schaan, Liechtenstein). One universal adhesive (iBond Universal, Heraeus Kulzer) was used for bonding procedures. Chemical compositions and materials’ handling are reported in Table 1.

**Table 1:** Chemical composition and mode of use of the materials used in the study

Material	Composition	Procedure
iBond Universal Adhesive (Heraeus Kulzer GmbH, Hanau, Germany. LOT: K010028)	MDP, 4-META, methacrylates, acetone, water	Drop the adhesive into the mixing well and use within 3 min. Gently rub adhesive onto the entire dental surface for 20 s. Carefully air-dry with an oil-free air flow until the adhesive film no longer moves. The surface must be visibly glossy. Polymerize for 10 s (wavelength of 440–480 nm; light output: > 550 mW/cm^2^)
iBond Ceramic Primer (Heraeus Kulzer. LOT: K010101)	Isopropanol/acetone-based solution of methacrylate monomers and silane	Measure out the desired drops of solution into the mixing well. Brush the whole surface to be treated without delay and allow to dry for 20 s. Dry briefly with an oil-free air-flow
Venus Diamond (Heraeus Kulzer; Shade: A2; LOT: K010069)	Light-curing, radiopaque nanocomposite. Barium aluminum boro fluorosilicate glass, TCD-urethane acrylate, silica, UDMA, TEGDMA, titanium dioxide, fluorescent pigments, metallic oxide pigments, organic pigments, aminobenzoic acid ester, camphoroquinone	Apply the material in thin layer (max 2 mm) and adapt to the cavity walls. Polymerize (wavelength of 440–480 nm; light output: > 550 mW/cm^2^)
RelyX Ultimate (3 M, St Paul, MN, USA; Shade: A1; LOT: 669,768)	Base paste: methacrylates monomers, radiopaque silanated fillers, initiator components, stabilizers, rheological additives. Catalyst paste: methacrylate monomers, radiopaque alkaline basic fillers, stabilizers, pigments, rheological additives, fluorescence dye, dark cure activator	For each application, a new mixing tip was used. Dispense the cement from the automix syringe and apply the desired quantity directly to the restoration. Excess were removed while seating in place the restoration
Variolink Esthetic DC (Ivoclar Vivadent; Shade: Neutral; LOT: W88206)	Monomer matrix: urethane dimethacrylate and further methacrylate monomers. Inorganic filler: ytterbium trifluoride and spheroid mixed oxide, initiators, stabilizers and pigments	For each application, a new mixing tip was adopted. Dispense the cement from the automix syringe and apply the desired quantity directly to the restoration. Seat the restoration and remove all the excess of the luting material

*MDP* methacryloyloxydecyl dihydrogen phosphate. *4-META* 4-methacryloyloxyethy trimellitate anhydride. *UDMA* urethane dimethacrylate. *TEGDMA* triethylene glycol dimethacrylate

The specimens were further randomly divided in 2 subgroups according to the polymerization mode of the resin cements (*N* = 5): self-cure mode (SC; 1 h at 37 °C) or dual-cure mode (DC; 20-s light-cure followed by 15 min of self-cure at 37 °C). A LED curing light was used (Demi™Plus, Kerr Dental). Luting procedures were performed under a sustained seating pressure of 1 kg maintained during the cementation procedures, until the complete polymerization of the cements both in the SC and DC modes. In the DC groups, light curing was performed from the top of the restoration, making sure to maintain the lamp tip as close as possible to the restoration surface.

Specimens were cut into resin-dentin sticks with cross-sectional area of ~ 0.9 mm^2^. The sticks were equally and randomly divided into two groups and stored in an incubator at 37 °C in artificial saliva for 24 h (*T*_0_) or 12 months (*T*_12_). After aging, each stick was measured with a digital caliper and stressed to failure under tension using a simplified universal testing machine (Bisco Inc., Schaumburg, IL, USA; crosshead speed: 1 mm/min). Both parts of the fractured sticks were observed under a stereomicroscope (Stemi 2000-C; Carl Zeiss Jena GmbH) to determine the mode of failure (50 ×): adhesive at the dentin (AD) or composite (AC) interface, cohesive in dentin (CD), cohesive in composite (CC), or mixed (M). Fractographic analysis was performed with scanning electron microscopy (SEM, Nova NanoSEM 450. Thermo Fisher Scientific, Waltham, MA, USA). The dentin side of the fractured sticks was mounted on metal stubs and gold sputter coated before evaluation at different magnifications (accelerating voltage of 10.00 kV and magnifications at 200 × and 500 ×).

After checking the normality (Kolmogorov–Smirnov) and the homoscedasticity (modified Levene’s test) of the data, a two-way analysis of variance (ANOVA) was run to evaluate the effects of the independent variables “resin cement” (RXU vs VAR) and “curing mode” (SC or DC) and the interactions of these factors on the µTBS. Tukey post hoc tests were used to determine differences among the groups. Premature debondings (failures occurred before testing) were included into the statistical analysis and calculated as zero bond values. Statistical significance was pre-set at *p* = 0.05.

### Nanoleakage expression

Additional specimens (*n* = 4 per group) were bonded as previously described for the µTBS test and sectioned into 1-mm-thick slices. The interfacial nanoleakage expression was quantified at baseline (*T*_0_) or after 12 months (*T*_12_) of laboratory storage at 37 °C. The specimens were immersed in 50 wt% ammoniacal silver nitrate solution for 24 h under laboratory light, and, after copious rinsing in distilled water, they were immersed into a photo-developing solution to reduce the silver ions into metallic silver grains. The specimens were fixed on glass slides using cyanoacrylate glue, flattened with SiC papers of increasing fineness (180-, 600-, 1200-, 2400-, and 4000-grit) and observed under a light microscope (Nikon E800; Nikon, Tokyo, Japan. Magnification: 20 ×) to analyze the precipitation of the silver grains along the bonded interfaces. The specimens were graded on a 0–4 scale according to the severity of interfacial nanoleakage and quantified by one experienced investigator [16]. The mean nanoleakage percentage of the specimens were averaged for statistical analysis using the non-parametric chi-square test (*p* = 0.05).

### In situ* zymography*

Middle/deep dentin (*N* = 4) was cut into 1-mm-thick slabs. Each slab was divided into four quarter slices to test all the experimental groups on the same substrate. A standardized smear layer was created on each quarter with #240 SiC paper. One surface of each quarter per slab was treated according to the bonding procedures used for the µTBS test. The bonded assemblies were sectioned vertically into 1-mm-thick specimens to expose the resin-dentin interfaces. Each specimen was then glued to glass slides, ground down to ~ 50 µm, polished and prepared for in situ zymography [17]. Briefly, a self-quenched fluorescein-conjugated gelatine mixture (E-12055; Molecular Probes, Eugene, OR, USA) was placed on top of each specimen to cover the resin-dentin interfaces, protected with a coverslip and incubated overnight in a dark humidified chamber at 37 °C. The specimens were examined with a confocal laser scanning microscope (excitation wavelength: 488/530 nm; LeicaSP8, TCS SP2 AOBS, Leica Microsystem GmbH, Wetzlar, Germany). For each assembly, a series of images were made (one image per each 1 µm into the depth of the sample) to show the hydrolysis of the quenched fluorescein-conjugated gelatine substrate, presented as green fluorescence. ImageJ software (National Institute of Health, Bethesda, MD, USA) was used to quantify integrated density of the fluorescence signals. Since the in situ zymography data were not normally distributed (Shapiro–Wilk test), the non-parametric Mann–Whitney *U* test was used to compare the density of fluorescence signal between two groups. The level of significance was set at *p* = 0.05.

## Results

### Microtensile bond strength test

Mean µTBS values, standard deviations, and percentage of failure modes are shown in Table 2. The curing mode statistically significantly influenced the µTBS of the tested materials, both immediately and after aging (*p* < 0.05). At *T*_0_, the DC resin cements recorded higher tensile strengths than those in the SC groups. RXU/DC attained the highest immediate bond strength results at *T*_0_ (*p* < 0.05). No differences in bond values were observed between the groups tested immediately in the SC mode (*p* = 0.05).

**Table 2:** Mean µTBS values** ± **standard deviations expressed as MPa. Different letters show statistically significant differences among groups in the row (*p* < 0.05). Capital letters indicate significant differences among groups in the column (*p* < 0.05). Numbers indicate statistically significant differences between *T*_0_ (specimens tested after 24 h) and *T*_12_ (specimens tested after storage for 12 months in artificial saliva at 37 °C) (*p* < 0.05). Percentages of failure modes after µTBS test are also shown. A common trend was the occurrence of mixed failures in all the experimental groups, irrespective of the storage condition. Cohesive failures in composite were only recorded for RXU at baseline, irrespective of the curing mode. After aging, an increase of adhesive failures was observed for RXU in both curing modes

Materials	RXU		VAR	
Curing mode	SC	DC	SC	DC
T_0_ (MPa)	23.8 ± 8.4 **C1**	40.8 ± 10.4 **A1**	23.3 ± 9.8 **C1**	33.3 ± 8.0 **B1**
	95% M; 5% CC	98% M; 2% CC	82%M; 18%AD	92% M; 8% AD
T_12_ (MPa)	20.8 ± 9.1 **b2**	26.6 ± 8.5 **a2**	14.4 ± 6.8 **c2**	28.5 ± 11.0**a2**
	88% M; 12% AD	97% M; 3% AC	93% M; 7% AC	100% M

Laboratory storage contributed to bond strength reduction for all the tested materials. However, at *T*_12_ the DC groups still yielded higher bond strength values than the SC groups (*p* < 0.05) regardless of the materials used. Differences were, however, present in the SC groups with RXU recording higher bond strengths than VAR (*p* < 0.05).

Mixed failures were the most frequent mode of failures in all groups, ranging between 82 and 100%, independent of the tested material, and with no differences at *T*_0_ or *T*_12_. Cohesive failures in composite were observed only for RXU at *T*_0_, both in SC (5%) and DC (2%) mode. At baseline, only VAR registered adhesive failures at the dentin side (18% in the SC and 8% in the DC groups, respectively). At *T*_12_, no cohesive fractures were observed among the groups tested, while there were adhesive failures at the dentin side in the RXU/SC group (12%) and adhesive at the composite interface in the RXU/DC (3%) and VAR/SC (7%) groups.

**Fig. 1:**
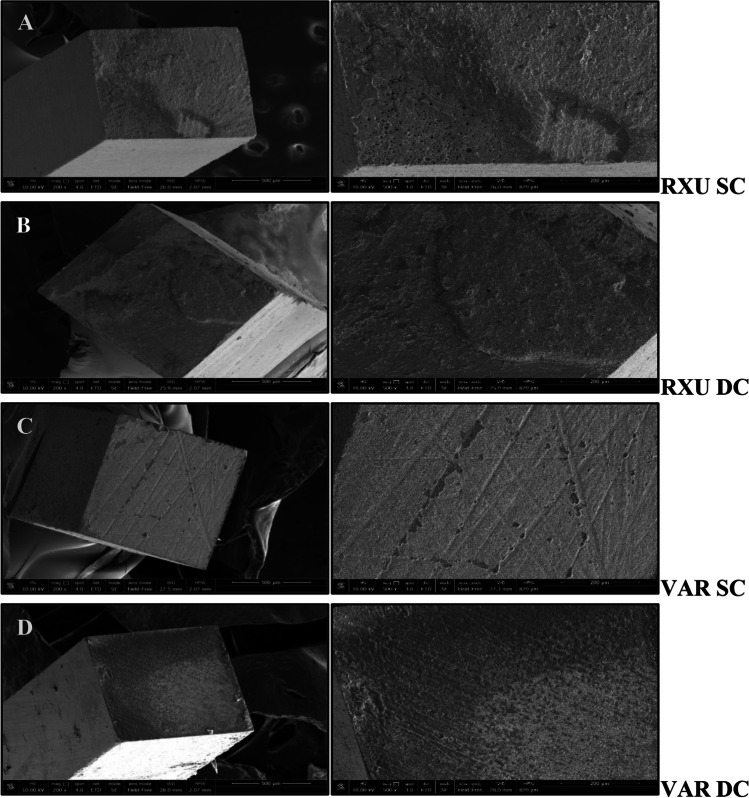
SEM microphotographs of representative fractured beams of the dentin side (200 × , image on the left) and details of the bonded surface (500 × , image on the right) of RelyX Ultimate (RXU) and Variolink EstheticDC (VAR) in the self-cure (SC) or dual-cure (DC) polymerization mode, after 24-h testing (*T*_0_). **A**–**B** RXU SC and DC, respectively; **C**–**D** VAR SC and DC, respectively

**Fig. 2:**
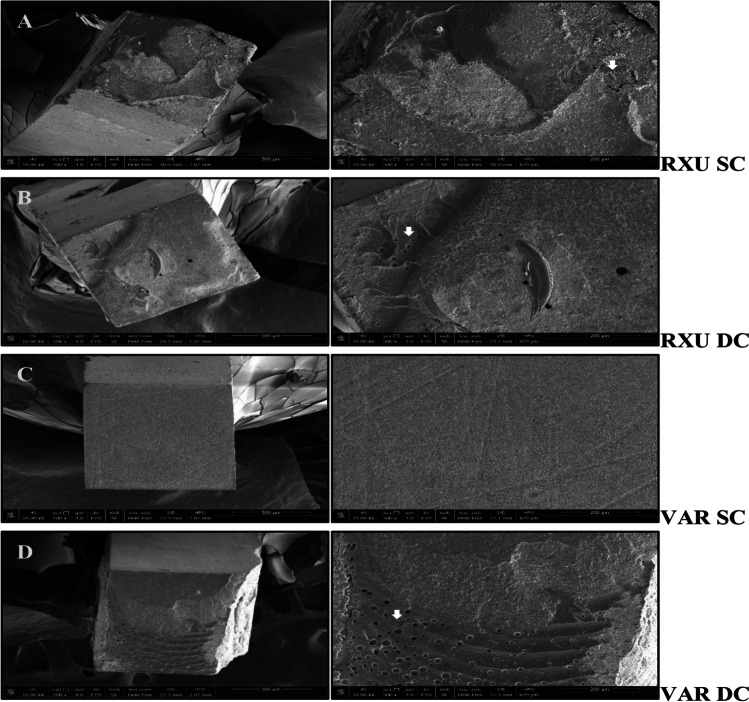
SEM microphotographs of representative fractured beams of the dentin side (200 × , image on the left) and details of the bonded surface (500 × , image on the right) of RelyX Ultimate (RXU) and Variolink EstheticDC (VAR) in the self-cure (SC) and dual-cure (DC) mode, after 12 months of laboratory aging (*T*_12_). **A**–**B** RXU SC and DC, respectively; **C**–**D** VAR SC and DC, respectively. Mixed adhesive/cohesive failure pattern was predominant in all tested groups. Rough surface with porous agglomerates were noticed, probably related to areas of suboptimal polymerization producing irregularities at the cement/dentin interfaces (white arrows). These sites may expedite premature restoration debonding

Figures 1 and 2 show SEM images of debonded beams at different magnifications (200 × image on the left, and 500 × image on the right), at baseline and after 1 year of artificial aging, respectively. At *T*_0_, structural defects were observed inside the cement bulk of RXU SC (Fig. 1A). These defects consisted in voids and bubbles that were absent in RXU DC, where the cement surface appeared more homogeneous and adherent to the tooth substrate, characterizing a pure cohesive failure within the cement (Fig. 1B). Open dentinal tubule orifices with no resin impregnation were detected for VAR SC, with traces of adhesive remnants attached to the tooth substrate (Fig. 1C). Surfaces of fractured VAR DC beams appeared rough with dentinal tubule sparsely occluded with resin (Fig. 1D).

After laboratory aging (T_12_), untight surfaces were still present for RXU SC (Fig. 2A) with compartmentalized defects and voids into the cement bulk when RXU was DC (Fig. 2B). Rough but more homogeneous surface characterized the adhesive interface of VAR SC with the presence of filamentous, short agglomerates (Fig. 2C). Flaws and voids were observed for VAR DC (Fig. 2D).

### Nanoleakage expression

**Fig. 3:**
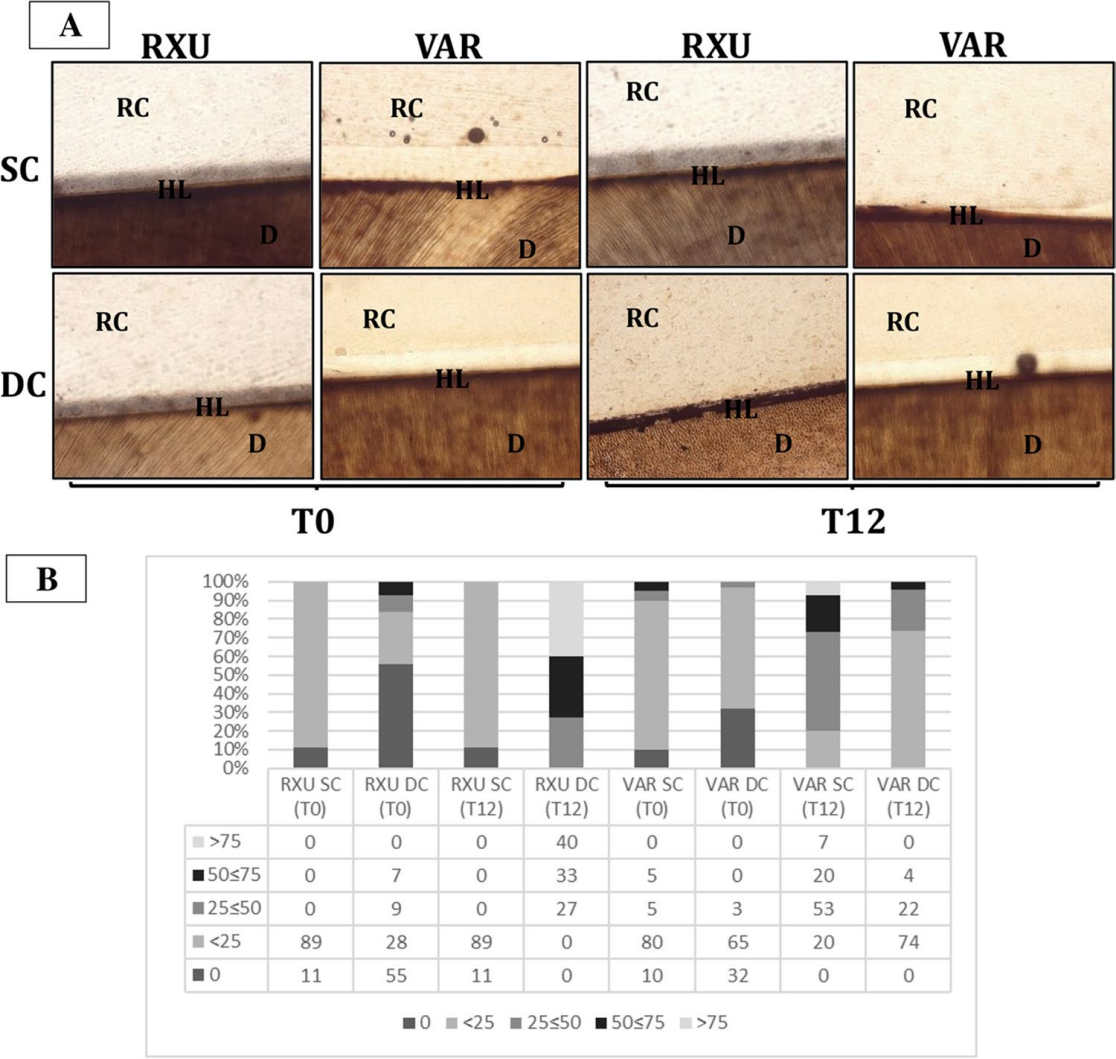
**A** Representative light microscopy images (100 × magnification) of the tested materials in the experimental conditions and submitted to nanoleakage with silver nitrate after 24 h (*T*_0_) and 12 months of artificial aging (*T*_12_). Higher amount of silver granules was accumulated when VAR was only self-cured. RXU/DC showed higher interfacial leakage after 1 year of aging. **B** Percentage of interfacial nanoleakage expression at the resin cement-dentin interfaces created for each tested group in the self-cure (SC) or dual-cure (DC) activation mode of the resin luting agent: RelyX Ultimate (RXU); Variolink EstheticDC (VAR). The materials were observed after 24 h (*T*_0_) or 12 months (*T*_12_) of aging in artificial saliva

Representative light microscopy images of the interfacial nanoleakage expression for the four experimental groups and descriptive statistics are presented in Fig. 3A–B, respectively. The chi-square test indicated that the curing mode (SC vs DC) and storage significantly influenced silver grains accumulation at the bonded interfaces (*p* < 0.05).

### In situ* zymography*

**Fig. 4:**
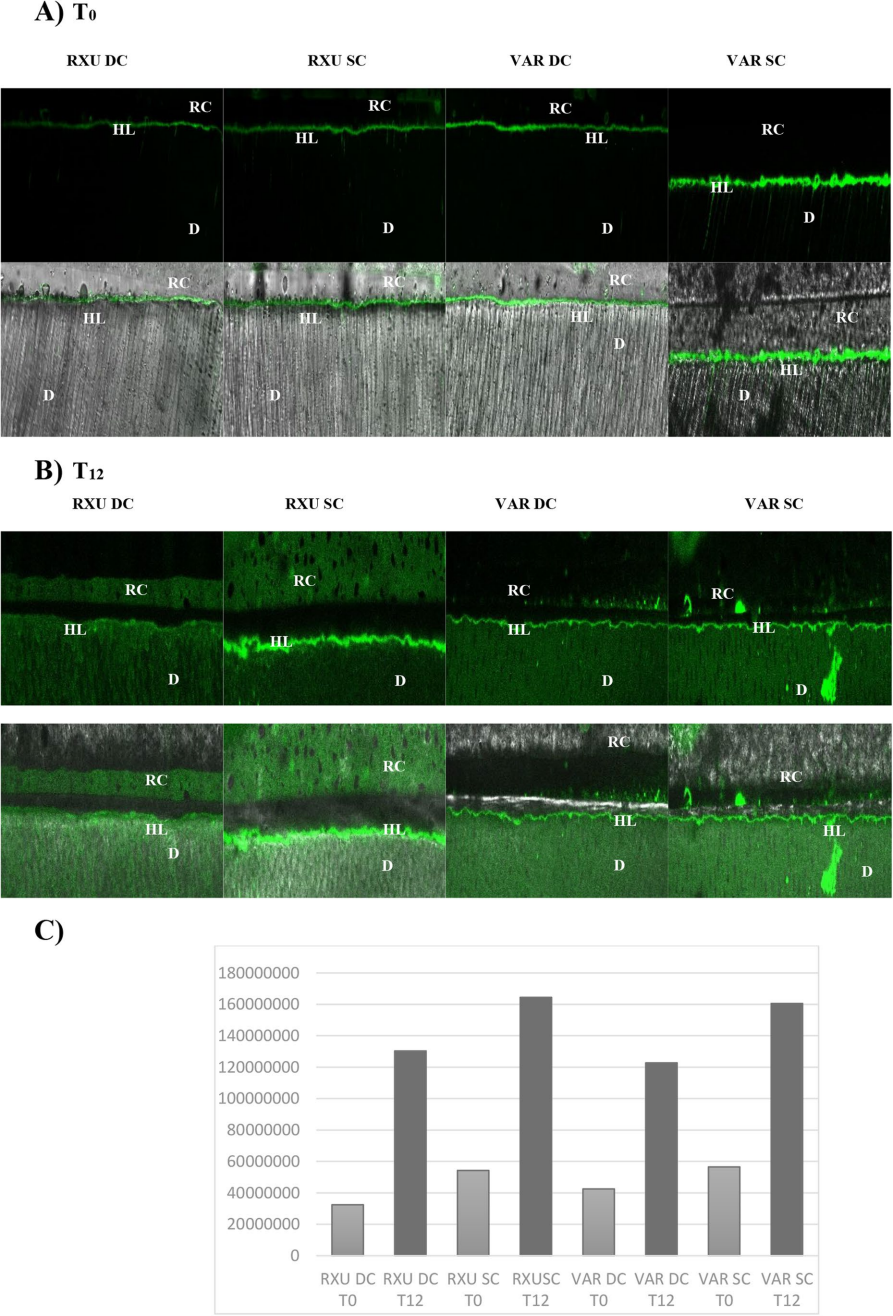
Resin-dentin interfaces incubated with quenched fluorescein-labeled gelatin. Images acquired in green channel, showing fluorescence (identifying intense endogenous enzymatic activity) in dentinal tubules and within the HL (top figures) and images obtained by merging differential interference contrast image (showing the optical density of the resin-dentin interface) and image acquired in green channel (bottom images). **A** Evaluation after 24 h showed higher fluorescence within the HL when the specimens were SC**. B** After 1-year aging, staining was noticed also within the cement bulk. The high permeability of the universal adhesive may have accounted for the amount of water emanating from the bonded interfaces, possibly causing a disruption in the HL and allowing gelatin to enter giving fluorescent signals in the cement. D, dentin; HL, hybrid layer; R, resin cement. **C** Statistical analysis indicated the “curing mode” and “aging” as the variable influencing the interfacial stability, with specimens in the SC mode after 1-year aging showing the higher fluorescence impregnation (*p* < 0.05)

Representative images of the gelatinolytic activity in the resin-dentin interfaces of the experimental groups are shown in Fig. 4. The results obtained on the confocal laser scanning microscope revealed higher fluorescence signal when the resin cements were SC, more evident after the storage period of 1 year (*p* < 0.05). SC specimens showed a disruption in the HL with some fluorescent signal in the cement bulk (Fig. 4C–D).

## Discussion

The results of this study revealed that light activation influences the immediate bonding performances and the endogenous dentinal enzymatic activity of the dual-cure resin cements tested, requiring the rejection of the first and second null hypothesis, respectively. After 1-year aging, a significant reduction in bond strength values, increased silver granules uptake at the adhesive interfaces and higher endogenous enzymatic activity within the hybrid layer were found when the materials were SC. Accordingly, also the third null hypothesis has to be rejected.

To the best of the authors’ knowledge, this is the first study aiming to morphologically characterize the adhesive interfaces according to the curing mode of the resin cements. The materials used in the study are user-friendly and versatile as they are intended to be used without an additional activation step. Two resin cements containing different photoinitiator systems, in two curing modes and combined with an MDP-containing, HEMA-free universal adhesive were used for the study. The selection of the adhesive system can influence the polymerization efficacy of dual-cure resin cements [18]. iBond Universal contains hydrophilic and hydrophobic functional monomers, able to bind simultaneously to hydrophobic composite surface and hydrophilic dentin substrate. Specifically, 4-methacryloyloxyethyl trimellitate anhydride (4-META) is able to dissolve the minerals contained in the hard dental tissues and bind to the calcium of hydroxyapatite with the carboxylic chains. On the other hand, the molecule of methacryloyloxydecyl dihydrogen phosphate (MDP) chemically binds to the calcium of the hydroxyapatite with the acidic phosphoric groups on one side, while on the other side the methacrylic groups establish strong interactions with the resin composite. The deposition of salts of MDP calcium at the adhesive interface increases its mechanical strength and protects against hydrolysis [19]. Notwithstanding the adhesive layer light activation prior to the resin cement application [6], when resin cements were SC, lowered adhesive performances were observed compared to the DC groups (Table 2). The chemical incompatibility due to the formation of an oxygen-inhibited layer containing dissolved acidic monomers [13, 14, 20], along with the slower SC polymerization of the cements [21] and the facilitated evaporation of the organic solvent [22] can negatively affect the polymerization reaction and influence the bond strength and endogenous enzymatic activity. Additionally, iBond has been previously demonstrated as highly sensitive to water seepage through dentin [23]. The accumulation of water at the adhesive interface can hamper the resin cement polymerization and impair the mechanical properties of the materials, thus promoting hydrolysis phenomena inside the cement and the breakdown of the polymer chains of the resin matrix, present in the form of defects or bubbles, which represent sites from which the stresses can dissipate and affect the durability of the bond [12, 24]. This was corroborated by SEM and confocal microscopy images that revealed a porous surface of the SC specimens (Figs. 1 and 2), hypothesized as a consequence of the water damage that caused a disruption into the HL. After aging, water uptake was increased permeating the cement pores along with fluorescent gelatin (Fig. 4B).

When dual-cured RXU and VAR were used in combination with their correlative adhesives (i.e., Scotchbond Universal and Adhese Universal, respectively), microtensile bond strength values were comparable to those obtained in this study [9]. Yet, after 1-year aging, the present results were even higher than those obtained after thermocycling [9].

The increased interaction demonstrated by RXU when the material was DC has been extensively accepted to be related to a higher degree of polymerization when compared to the SC [6, 25]. Moreover, when RXU was used in association with an MDP-containing adhesive, the deposition of MDP and calcium on tooth substrates resulted in higher bond strengths [19]. However, in the present study, the interfacial nanoleakage analysis revealed higher silver grains deposition (Fig. 3) and increased endogenous enzymatic activity after storage (Fig. 4), confirming that the interaction of dual-cure resin cements with acidic functional monomers can hamper the polymerization behavior [14, 26]. Dual-cure resin cements are expected to provide high degree of conversion and increased mechanical properties compared to the SC mode [7, 27]. Many factors may cause the reduction of light intensity, such as the presence of indirect restorations that provide true obstacles for the penetration of light transmission. When the resin cement is light-cured through the interposition of an indirect restoration, only 55–75% of the degree of conversion (DC) can be obtained, depicting a directly proportional relationship between the degree of polymerization and mechanical properties [28]. Composition, opacity, thickness, and shade of the prosthetic restoration attenuate the intensity of light and reduce the number of photons reaching resin cement possibly compromising the prognosis of the restoration [6, 29]. The light energy in the photo-polymerization mechanism should result in rapid setting of the resin matrix, allowing little or no possibility for adverse acid–base reactions to occur [13]. Despite the dual-polymerization mechanism, the self-cure components are not able to compensate the low light irradiance decreased in the presence of the composite overlay nor are as effective in producing monomer conversion [30]. The interaction between unreacted acidic monomers and the remnants of the photo-initiators would impede the participation of amines in the redox process, resulting in the failure of the polymerization reaction [12, 13]. The seepage of water from the underlying dentin through the adhesive interfaces may enable the formation of swallowed cement bulk and bubbles (Figs. 1 and 2) [31].

The shade of the cement might also influence the mechanical and optical properties of the luting material [29]. The hardness of the resin was shown to be inversely proportional to the darker shade of the resin cement demonstrating that a specific activation strategy is essential for each cement shade in order to maximize the material’s mechanical properties [32]. Transparent resin cements enable higher depth of cure and microhardness values due to their ability to absorb more light than the opaque cement [32]. In the present study, lighter values (A1 shade for RXU and neutral shade for VAR) of the resin cements were used to reduce the influence of color shade.

When VAR was DC, it attained higher bond strengths than when it was only SC, both at *T*_0_ and *T*_12_. The photoinitiator contained in the VAR (Ivocerin) showed high reactivity to curing light and allowed an efficient polymerization boost at a depth of 4 mm [33] even in the presence of an indirect restoration. However, concerns have been raised on the solubility of the material, as leaching of unreacted monomers out of the material was observed [34]. Open dentinal tubules were evidenced at the adhesive interface (Figs. 1–2) possibly due to the inability of the adhesive to fully infiltrate the exposed collagen mesh, and the incomplete evaporation of the solvent (acetone, in the case of iBond universal), which negatively influenced the bonding performances [35]. If not properly polymerized, the high acidic monomer content at the adhesive layer can interfere with the free radical formation promoted by the photoinitiator, limiting the polymerization reaction even when DC [36].

After 1 year of artificial aging, in situ zymography further confirmed that light activation is a fundamental step when dealing with dual-cured resin-based cements to diminish the HL degradation. This should be taken into consideration during the cementation of thick and opaque restorations. In this context, photoactivation should be considered an important aspect in the achievement of clinical success. The tested cements are amine-free but differ in terms of chemical composition and photoactivator systems. Photoinitiators alternative to camphorquinone, such as those contained in RXU and VAR, have been developed over time to overcome the “yellowing effect” in dental resins. These photoinitiators possess different light absorptivity and can generate free radicals through a direct cleavage process without the need of co-initiators [37]. Despite their satisfactory esthetic properties and curing efficiency, they were demonstrated to be very sensitive to light attenuation [38].

Within the intrinsic limitations, the results obtained in the present in vitro study indicated that light activation is still a fundamental step when dealing with dual-cured resin-based cements, confirming that the influence of the light exposure on the polymerization process is material dependent. Further studies (such as spectroscopy analysis) are necessary to obtain quantitative information on the influence of the activation mode on resin cement polymerization when resin-based luting materials are used in combination with different universal adhesives.

## Conclusions

Despite the dual-polymerization mechanism, the self-cure components are not able to compensate the lower light irradiance decreased in presence of the composite overlay nor are as effective in producing monomer conversion. Light-curing preserved the microtensile bond strength and decreased the nanoleakage expression and endogenous enzymatic activity of the tested dual-cure resin cements.
